# Supplementary material for: The sensitivity of a radical pair compass magnetoreceptor can be significantly amplified by radical scavengers
Source: Sci Rep. 2017 Sep 14;7:11640. doi: 10.1038/s41598-017-09914-7 (PMC5599710; doi:10.1038/s41598-017-09914-7)
Supplement: Supplementary file 1 — Supporting Information [file 41598_2017_9914_MOESM1_ESM.pdf]

The sensitivity of a radical pair compass magnetoreceptor can be significantly amplified by radical scavengers

Daniel R. Kattnig† and P. J. Hore*

Department of Chemistry, University of Oxford, Physical and Theoretical Chemistry Laboratory, South Parks Road, Oxford OX1 3QZ, U.K.

* Author for correspondence: peter.hore@chem.ox.ac.uk
† Permanent address: Department of Physics, University of Exeter, Living Systems Institute, Stocker Road, Exeter, EX4 4QD

Appendix

Singlet-triplet interconversion in AB

Signalling state anisotropy

Supporting Information

Figure S1: Anisotropic yields of the signalling state, S, and the scavenging product, X, for a model [$FAD^{\bullet-}$ $WH^{\bullet+}$] radical pair.

Figure S2: Anisotropic yields of the signalling state, S, and the scavenging product, X, for a model [$FAD^{\bullet-}$ $WH^{\bullet+}$] radical pair.

Figure S3: Anisotropic yields of the scavenging product, X, for a model [$FAD^{\bullet-}$ $WH^{\bullet+}$] radical pair.

Figure S4: Anisotropic yields of the scavenging product, X, for various model radical pairs.

Figure S5: Anisotropic yields of the signalling state, S.

Figure S6: Anisotropic yields of the signalling state, S.

Section S1: Derivation of Eq. (3).

Section S2: Derivation of Eqs (6) and (8).

Appendix

Singlet-triplet interconversion in AB

Insight into the origin of singlet-triplet interconversion in AB as a result of a spin-selective AC scavenging reaction may be obtained from the following simple argument.[36]

First, we define the usual singlet and triplet states for the AB and AC pairs:

$$\begin{aligned} \left|\mathrm{S}^{\mathrm{AX}}\right\rangle &= \frac{1}{\sqrt{2}}\left|\alpha^{\mathrm{A}}\beta^{\mathrm{X}}\right\rangle - \frac{1}{\sqrt{2}}\left|\beta^{\mathrm{A}}\alpha^{\mathrm{X}}\right\rangle \\ \left|\mathrm{T}_{+1}^{\mathrm{AX}}\right\rangle &= \left|\alpha^{\mathrm{A}}\alpha^{\mathrm{X}}\right\rangle \\ \left|\mathrm{T}_{0}^{\mathrm{AX}}\right\rangle &= \frac{1}{\sqrt{2}}\left|\alpha^{\mathrm{A}}\beta^{\mathrm{X}}\right\rangle + \frac{1}{\sqrt{2}}\left|\beta^{\mathrm{A}}\alpha^{\mathrm{X}}\right\rangle \\ \left|\mathrm{T}_{-1}^{\mathrm{AX}}\right\rangle &= \left|\beta^{\mathrm{A}}\beta^{\mathrm{X}}\right\rangle, \end{aligned} \tag{1}$$

where X = B or C. We start with the AB pair in its singlet state, $\left|\mathrm{S}^{\mathrm{AB}}\right\rangle$, and the radical C in state $\left|\alpha^{\mathrm{C}}\right\rangle$ ($\left|\mathrm{S}^{\mathrm{AB}}\beta^{\mathrm{C}}\right\rangle$ is considered below). This initial state, $\left|\psi\right\rangle = \left|\mathrm{S}^{\mathrm{AB}}\alpha^{\mathrm{C}}\right\rangle$ in the $\{\mathrm{AB}\}\mathrm{C}$ basis, can be expressed in the ABC product basis using equations (1):

$$\left|\psi\right\rangle = \frac{1}{\sqrt{2}}\left|\alpha^{\mathrm{A}}\beta^{\mathrm{B}}\alpha^{\mathrm{C}}\right\rangle - \frac{1}{\sqrt{2}}\left|\beta^{\mathrm{A}}\alpha^{\mathrm{B}}\alpha^{\mathrm{C}}\right\rangle, \tag{2}$$

and then transformed into the $\{\mathrm{AC}\}\mathrm{B}$ basis, again using equations (1):

$$\left|\psi\right\rangle = \frac{1}{2}\left|\mathrm{S}^{\mathrm{AC}}\alpha^{\mathrm{B}}\right\rangle - \frac{1}{2}\left|\mathrm{T}_{0}^{\mathrm{AC}}\alpha^{\mathrm{B}}\right\rangle + \frac{1}{\sqrt{2}}\left|\mathrm{T}_{+1}^{\mathrm{AC}}\beta^{\mathrm{B}}\right\rangle. \tag{3}$$

Equation (3) shows that the AC pair is 25% singlet and 75% triplet, as would be expected from the absence of correlation between C and either A or B.

Now we allow the AC singlets to recombine via a spin-selective scavenging reaction. To see the effect most clearly, we simply remove the first term on the right hand side of equation (3) to give the modified state $\left|\psi'\right\rangle$:

$$\left|\psi'\right\rangle = -\frac{1}{2}\left|\mathrm{T}_{0}^{\mathrm{AC}}\alpha^{\mathrm{B}}\right\rangle + \frac{1}{\sqrt{2}}\left|\mathrm{T}_{+1}^{\mathrm{AC}}\beta^{\mathrm{B}}\right\rangle. \tag{4}$$

$\left|\psi'\right\rangle$ can be transformed back into the ABC product basis:

$$\left|\psi'\right\rangle = -\frac{1}{2\sqrt{2}}\left|\alpha^{\mathrm{A}}\alpha^{\mathrm{B}}\beta^{\mathrm{C}}\right\rangle + \frac{1}{\sqrt{2}}\left|\alpha^{\mathrm{A}}\beta^{\mathrm{B}}\alpha^{\mathrm{C}}\right\rangle - \frac{1}{2\sqrt{2}}\left|\beta^{\mathrm{A}}\alpha^{\mathrm{B}}\alpha^{\mathrm{C}}\right\rangle, \tag{5}$$

and then into the original $\{\mathrm{AB}\}\mathrm{C}$ basis:

$$\left|\psi'\right\rangle = \frac{3}{4}\left|\mathrm{S}^{\mathrm{AB}}\alpha^{\mathrm{C}}\right\rangle + \frac{1}{4}\left|\mathrm{T}_{0}^{\mathrm{AB}}\alpha^{\mathrm{C}}\right\rangle - \frac{1}{2\sqrt{2}}\left|\mathrm{T}_{+1}^{\mathrm{AB}}\beta^{\mathrm{C}}\right\rangle. \tag{6}$$

Renormalizing $|\psi'\rangle$ gives:

$$|\bar{\psi}'\rangle = \frac{\sqrt{3}}{2}|S^{AB}\alpha^{C}\rangle + \frac{1}{2\sqrt{3}}|T_0^{AB}\alpha^{C}\rangle - \frac{1}{\sqrt{6}}|T_{+1}^{AB}\beta^{C}\rangle . \tag{7}$$

The proportions of singlet and triplet AB pairs, which were initially 100% and 0% respectively, are now 75% and 25%. If we start with $|\psi\rangle = |S^{AB}\beta^{C}\rangle$ instead of $|S^{AB}\alpha^{C}\rangle$, the equivalent of equation (7) is:

$$|\bar{\psi}'\rangle = \frac{\sqrt{3}}{2}|S^{AB}\beta^{C}\rangle - \frac{1}{2\sqrt{3}}|T_0^{AB}\beta^{C}\rangle + \frac{1}{\sqrt{6}}|T_{-1}^{AB}\alpha^{C}\rangle \tag{8}$$

which again gives 75% singlet and 25% triplet. Thus the net effect of the spin-selective AC reaction is to induce singlet-triplet interconversion in the AB pair even though there is initially no spin correlation between C and either A or B.

Signalling state anisotropy

The principal factor behind the unexpectedly large values of Δ_S and Γ_S appears to be the form of the hyperfine interactions of the N5 and N10 nitrogens in $FAD^{\bullet-}$. As the most anisotropic hyperfine interactions in the flavin radical, they seem to reinforce one another and to dominate the spin dynamics of $FAD^{\bullet-}$-containing radical pairs.[34] Both ^{14}N hyperfine tensors have almost perfect axial symmetry, with parallel symmetry axes, large *z*-components and near-zero *x*- and *y*-components.[11, 34] A consequence is that when the magnetic field is parallel to the symmetry axis, the spin Hamiltonian connects the AB singlet state to T_0^{AB} but not to T_{+1}^{AB} or T_{-1}^{AB}. By contrast, when the field is perpendicular to the hyperfine symmetry axis, S^{AB} is mixed with all three AB triplet states.

In the parallel configuration, the $S^{AB} \leftrightarrow T^{AB}$ interconversion caused by the AC scavenging reaction (see the Appendix in the main text) leads to T^{AB} states which (a) cannot be converted to S^{AB} by the spin Hamiltonian and are therefore unable to return to the ground state, (b) are not scavenged because of the Wigner spin-conservation requirements, and which therefore (c) contribute to a high yield of the non-selectively formed signalling state. It is these states that are responsible for the long-time behaviour shown in Fig. 3b (main text).

In the perpendicular case, the more extensive $S^{AB} \leftrightarrow T^{AB}$ mixing means that no T^{AB} states are immune to spin-selective recombination and scavenging. The result is a lower yield of the competing reaction that leads to the signalling state. Intermediate orientations show similar behaviour. It is only when the field is parallel to the dominant hyperfine axis that singlet-triplet mixing in the AB pair becomes restricted and the spike emerges. This qualitative difference between parallel and all other directions of the magnetic field seems to be responsible for the large anisotropies in the yield of the signalling state.

Figure S1

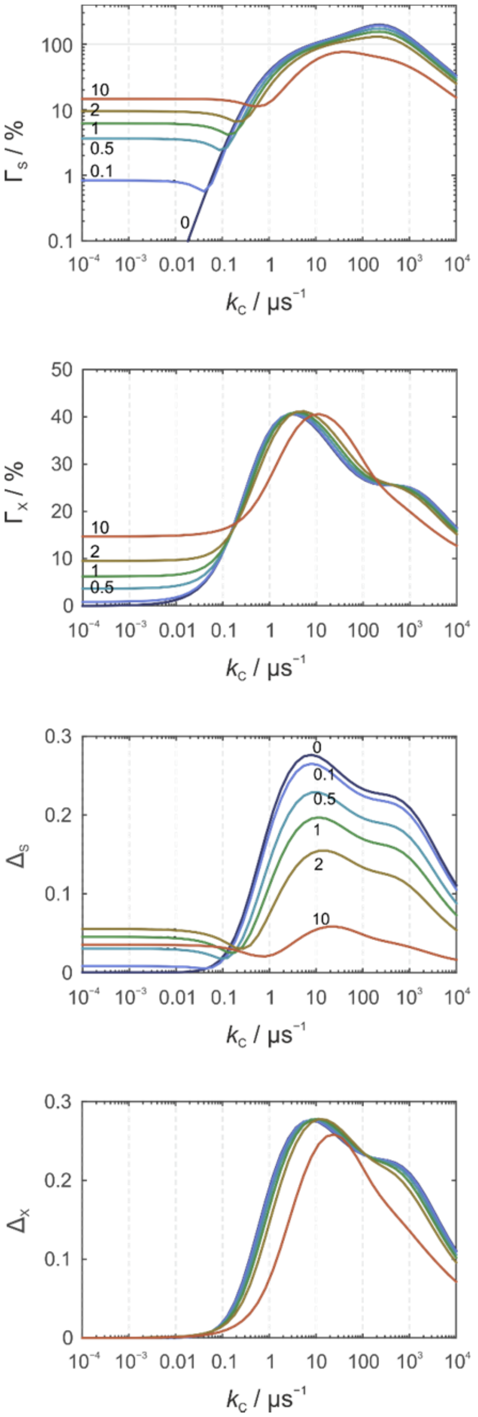

Anisotropic yields of the signalling state, S, and the scavenging product, X, for a model [$FAD^{\bullet-}$ $WH^{\bullet+}$] radical pair. The scavenger is a radical (J = ½) with no hyperfine interactions. (a) and (b) relative anisotropies (Γ_S and Γ_X, respectively), (c) and (d) absolute anisotropies (Δ_S and Δ_X, respectively), both as a function of the scavenging rate constant, k_C, for various values of ϕ. The spin system comprises N5 and N10 in $FAD^{\bullet-}$ and N1 in $WH^{\bullet+}$. The model is identical to that used for Figures 4(c) and 4(d) except that the scavenger reacted with $W^{\bullet+}$ instead of $FAD^{\bullet-}$. Γ_X and Δ_X are defined by analogy with Γ_X and Δ_X (equations (15) and (14), respectively).

Figure S2

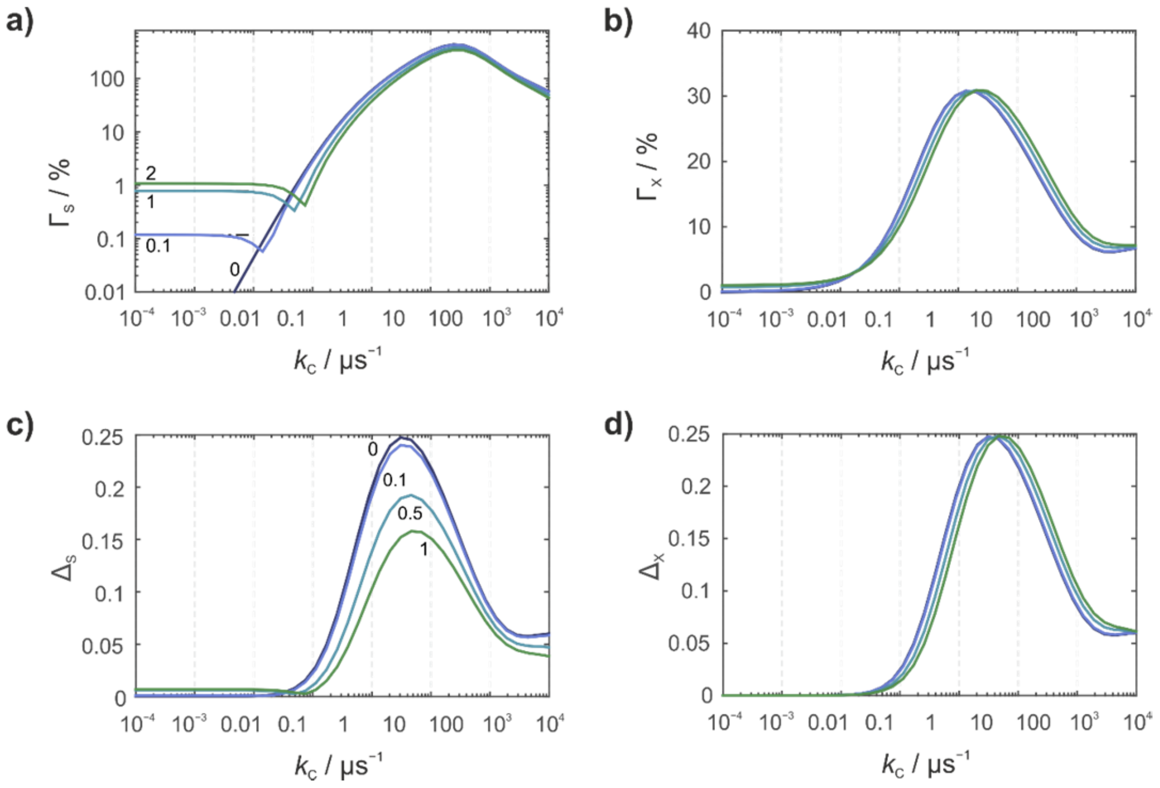

Anisotropic yields of the signalling state, S, and the scavenging product, X, for a model [FAD$^{•-}$ WH$^{•+}$] radical pair. The scavenger is a radical (J = ½) with no hyperfine interactions. (a) and (b) relative anisotropies (Γ_S and Γ_X, respectively), (c) and (d) absolute anisotropies (Δ_S and Δ_X, respectively), both as a function of the scavenging rate constant, k_C, for various values of ϕ. The spin system comprises N5, N10 and H6 in FAD$^{•-}$ and N1, H1, H4, Hβ and H7 in WH$^{•+}$. The scavenger reacted with FAD$^{•-}$.

Figure S3

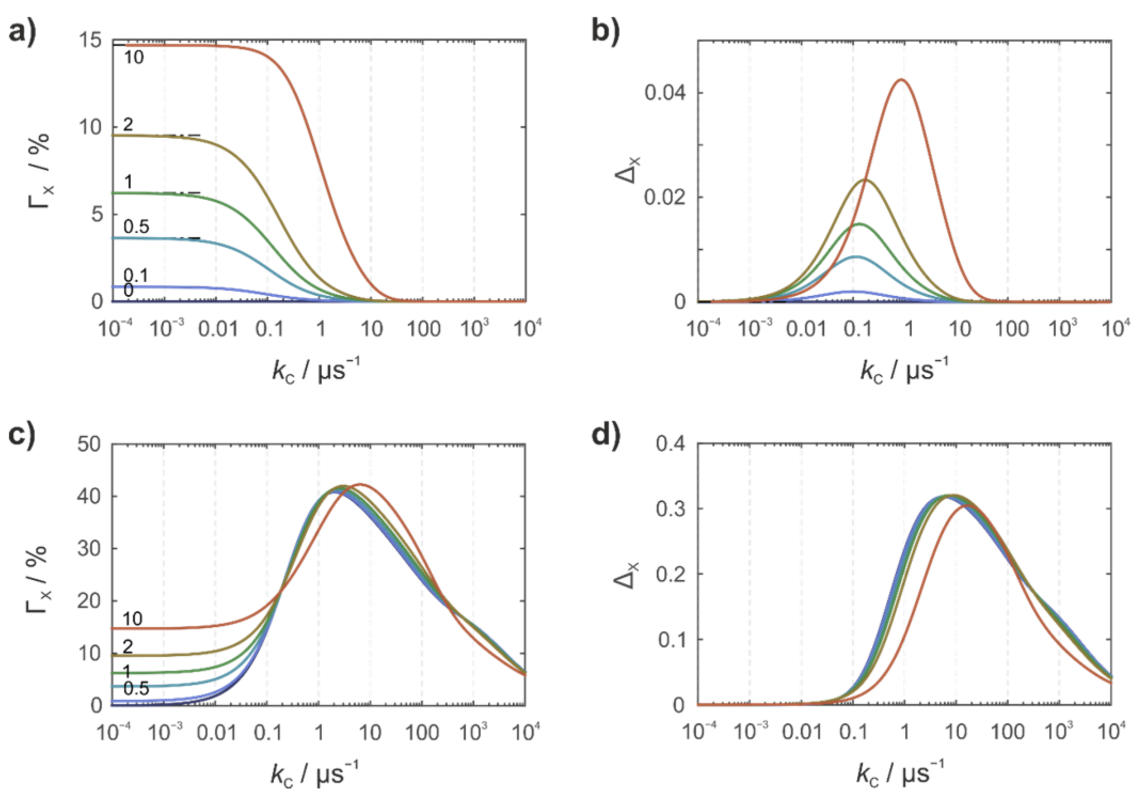

Anisotropic yields of the scavenging product, X, for a model [FAD$^{\bullet-}$ WH$^{\bullet+}$] radical pair. (a) and (c) relative anisotropies (Γ_X), (b) and (d) absolute anisotropies (Δ_X), both as a function of the scavenging rate constant, k_C, for various values of ϕ. In (a) and (b) the scavenger is diamagnetic (J = 0); in (c) and (d) it is a radical (J = ½) with no hyperfine interactions. The spin system comprises N5 and N10 in FAD$^{\bullet-}$ and N1 in WH$^{\bullet+}$. The scavenger reacted with FAD$^{\bullet-}$.

Figure S4

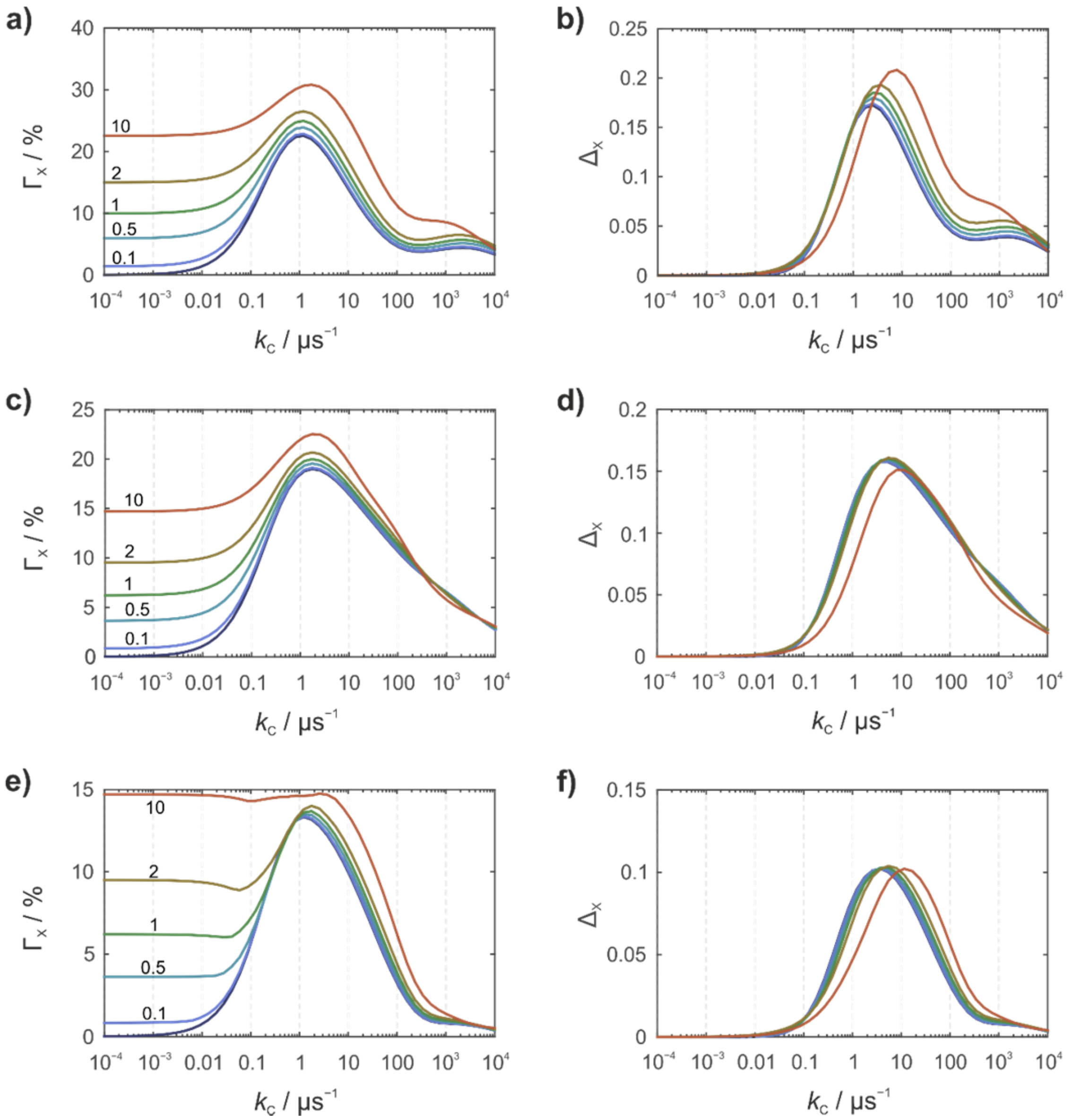

Anisotropic yields of the scavenging product, X, for various model radical pairs. (a) and (b) [FAD$^{\bullet-}$ Z$^{\bullet}$] radical pair with N5, N10, H6, H8 and Hβ in FAD$^{\bullet-}$ and no hyperfine interactions in Z$^{\bullet}$ or the scavenger. (c) and (d) [FAD$^{\bullet-}$ WH$^{\bullet+}$] radical pair with N5 and N10 in FAD$^{\bullet-}$ and N1 in WH$^{\bullet+}$. The scavenger had a single isotropic ^{1}H hyperfine interaction equal to that of the H4 proton in the ascorbyl anion radical[40]. (e) and (f) [FAD$^{\bullet-}$ WH$^{\bullet+}$] radical pair with N5 and N10 in FAD$^{\bullet-}$ and N1 in WH$^{\bullet+}$. The scavenger, which reacted with WH$^{\bullet+}$, was modelled on FAD$^{\bullet-}$ and included the N5 and N10 hyperfine interactions. These calculations are identical to those used for Figure 5 except that Γ_X and Δ_X are shown instead of Γ_S and Δ_S.

Figure S5

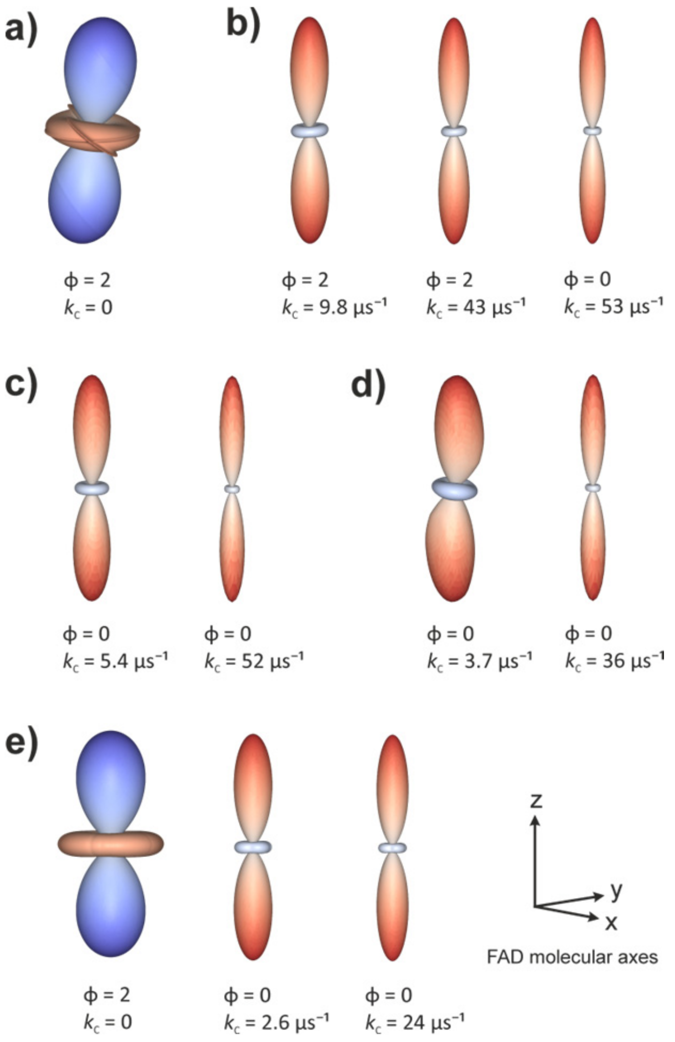

Anisotropic yields of the signalling state, S. (a) [$FAD^{\bullet -}$ $W^{\bullet +}$] radical pair with N5 and N10 in $FAD^{\bullet -}$ and N1 in $W^{\bullet +}$ and no scavenging reaction. (b), (c) and (d) show the results of the same calculation in the presence of scavenging by either (b) a radical with no hyperfine interactions, or (c) the ascorbyl radical model, or (d) another $FAD^{\bullet -}$ radical. (e) [$FAD^{\bullet -}$ $Z^{\bullet}$] radical pair with 7 nuclear spins in $FAD^{\bullet -}$ and none in $Z^{\bullet}$. Details of the model are discussed in the main text. The values of the parameters k_C and ϕ were chosen so as to show the anisotropy in the absence of the scavenging reaction or the anisotropy corresponding to the maximum Δ_S or the maximum Γ_S as a function of k_C (see Figures 4 and 5). In these plots the distance in any direction from the centre of each pattern to the surface is proportional to $\left|Y_S(\Omega)-\langle Y_S\rangle\right|$ when the magnetic field has that direction. Red/blue regions correspond to reaction yields larger/smaller than the average.

Figure S6

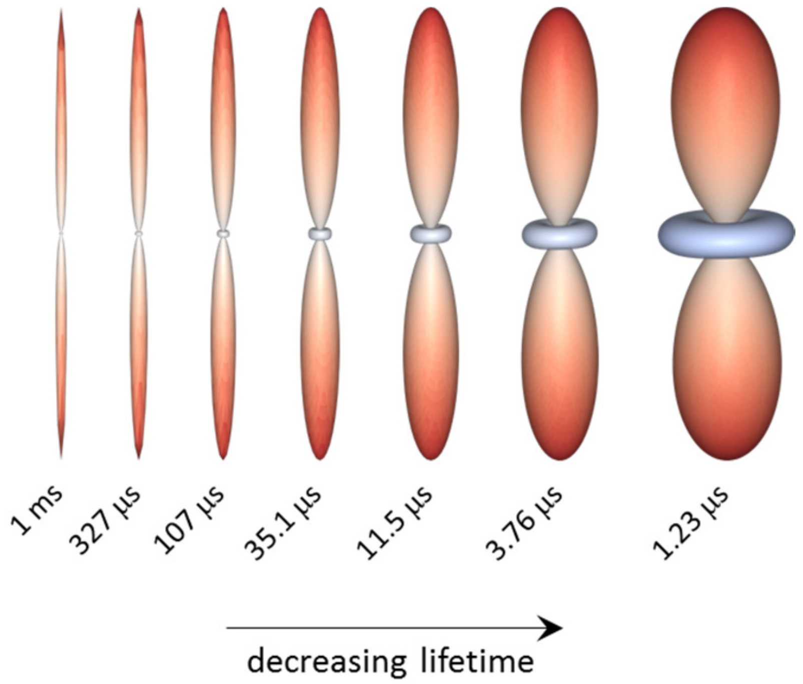

Anisotropic yields of the signalling state, S. [$FAD^{\bullet-}$ $WH^{\bullet+}$] radical pair with N5 and N10 in $FAD^{\bullet-}$ and N1 in $WH^{\bullet+}$. The scavenger, which reacted with $WH^{\bullet+}$, is a radical with no hyperfine interactions. $k_f = k_b$ with k_f^{-1} = 1000, 327, 107, 35.1, 11.5, 3.76, and 1.23 μs as shown. k_C = 65.1, 74.8, 79.2, 71.0, 49.6, 35.8, and 35.0 μs^{-1}, respectively, corresponding to the maximum anisotropy (Γ_S) for each value of k_f. The anisotropic yields were rescaled to reveal most clearly the increase in spikiness as the lifetime of the radical pair was prolonged. If drawn to scale, the pattern for k_f^{-1} = 1 ms would be 64 times taller than that for k_f^{-1} = 1.23 μs. See Figure S5 for a description of this type of plot.

S1. Derivation of Eq. (3)

Consider a doublet (S_A = ½) interacting with a particle with spin S_C = J. According to the Clebsch-Gordan series, the tensor product states associated with $\hat{\mathbf{S}}_A$ and $\hat{\mathbf{S}}_C$ can be combined to give eigenstates of the total angular momentum $\hat{\mathbf{S}}_{AC} = \hat{\mathbf{S}}_A + \hat{\mathbf{S}}_C$ with quantum numbers $J \pm \frac{1}{2}$. The projection operator for the $J - \frac{1}{2}$ case is proportional to

$$\hat{P}_{AC}^{-} \propto \hat{S}_{AC}^2 - (J+\tfrac{1}{2})(J+\tfrac{3}{2})\hat{1}, \qquad \text{(S.1)}$$

because any state with the complementary total angular momentum quantum number $J + \frac{1}{2}$ will be an eigenstate of $\hat{S}_{AC}^2$ with eigenvalue $(J+\frac{1}{2})(J+\frac{3}{2})$ and thus annihilated by the term proportional to $\hat{1}$. Eq. (S.1) can be simplified by expanding $\hat{S}_{AC}^2 = \left(\hat{\mathbf{S}}_A + \hat{\mathbf{S}}_C\right)^2 = \hat{S}_A^2 + \hat{S}_C^2 + 2\hat{\mathbf{S}}_A \cdot \hat{\mathbf{S}}_C$ and replacement of $\hat{S}_A^2$ and $\hat{S}_C^2$ by their respective eigenvalues multiplied by $\hat{1}$:

$$\hat{P}_{AC}^{-} = \frac{1}{N^{-}}\left(2\hat{\mathbf{S}}_A \cdot \hat{\mathbf{S}}_C - J\hat{1}\right). \qquad \text{(S.2)}$$

Here, N^{-} is a normalization constant to ensure that $\left(\hat{P}_{AC}^{-}\right)^2 = \hat{P}_{AC}^{-}$. An analogous argument suggests that

$$\hat{P}_{AC}^{+} = \frac{1}{N^{+}}\left(\hat{S}_{AC}^2 - (J-\tfrac{1}{2})(J+\tfrac{1}{2})\hat{1}\right) = \frac{1}{N^{+}}\left(2\hat{\mathbf{S}}_A \cdot \hat{\mathbf{S}}_C + (J+1)\hat{1}\right), \qquad \text{(S.3)}$$

The normalization constant can be established by requiring that $\hat{P}_{AC}^{+} + \hat{P}_{AC}^{-} = \hat{1}$, as the two total angular momentum states are mutually exclusive and complete. This yields

$$N^{+} = -N^{-} = 2J+1 \qquad \text{(S.4)}$$

which when combined with eqs (S.2) and (S.3) gives eq. (3).

S2. Derivation of Eqs (6) and (8)

For the sake of clarity, we focus on the case $k_C^+ = 0$. The general results given in eqs (6) and (8) may be derived in a similar fashion or obtained from the $k_C^+ = 0$ result by substituting $k_C^- \to k_C^- - k_C^+$ and multiplying the resulting expression by $\exp\left(-k_C^+ t\right)$.

Integrating eq. (4) for $k_C^+ = 0$, we obtain:

$$\begin{aligned}\hat{\rho}(t) &= \frac{1}{\mathrm{Tr}\left[\hat{P}_{AB}^{S}\right]}\exp\left(-\tfrac{1}{2}k_C^-\hat{P}_{AC}^-\right)\hat{P}_{AB}^{S}\exp\left(-\tfrac{1}{2}k_C^-\hat{P}_{AC}^-\right)\\ &= \frac{1}{\mathrm{Tr}\left[\hat{P}_{AB}^{S}\right]}\exp\left(-\tfrac{1}{2J+1}k_C^-\left(\tfrac{J}{2}-\hat{\mathbf{S}}_A\cdot\hat{\mathbf{S}}_C\right)\right)\left(\tfrac{1}{4}-\hat{\mathbf{S}}_A\cdot\hat{\mathbf{S}}_B\right)\exp\left(-\tfrac{1}{2J+1}k_C^-\left(\tfrac{J}{2}-\hat{\mathbf{S}}_A\cdot\hat{\mathbf{S}}_C\right)\right).\end{aligned} \tag{S.5}$$

The exponential terms are diagonal in a coupled representation of $\hat{\mathbf{S}}_A$ and $\hat{\mathbf{S}}_C$, while the term derived from $\hat{\rho}(0) \propto \hat{P}_{AB}^{S}$ is diagonal in the coupled representation of $\hat{\mathbf{S}}_A$ and $\hat{\mathbf{S}}_B$. We evaluate $\mathrm{Tr}\left[\hat{\rho}(t)\right]$ in the basis of the *coupled* eigenstates of the total angular momentum of $\hat{\mathbf{S}} = \hat{\mathbf{S}}_A + \hat{\mathbf{S}}_B + \hat{\mathbf{S}}_C$, $\hat{\mathbf{S}}_{AC} = \hat{\mathbf{S}}_A + \hat{\mathbf{S}}_C$ and $\hat{\mathbf{S}}_B$, i.e. the set of states $\left|((S_A,S_C)S_{AC},S_B)S,M\right\rangle \equiv \left|(S_{AC},S_B)S,M\right\rangle$. This yields

$$\hat{\rho}(t) = \frac{1}{\mathrm{Tr}\left[\hat{P}_{AB}^{S}\right]}\sum_{S_{AC}=J-\frac{1}{2}}^{J+\frac{1}{2}}\ \sum_{S=\left|S_{AC}-\frac{1}{2}\right|}^{S_{AC}+\frac{1}{2}}\ \sum_{M=-S}^{S} A^2(S_{AC})\left\langle(S_{AC},S_B)S,M\right|\hat{P}_{AB}^{S}\left|(S_{AC},S_B)S,M\right\rangle, \tag{S.6}$$

where

$$\begin{aligned}A(S_{AC}) &= \left\langle(S_{AC},S_B)S,M\right|\exp\left(-\tfrac{1}{2J+1}k_C^-\left(\tfrac{J}{2}-\hat{\mathbf{S}}_A\cdot\hat{\mathbf{S}}_C\right)\right)\left|(S_{AC},S_B)S,M\right\rangle\\ &= \exp\left(-\tfrac{1}{4J+2}k_C^-\left(J+S_A(S_A+1)+S_C(S_C+1)-S_{AC}(S_{AC}+1)\right)\right)\end{aligned} \tag{S.7}$$

which is independent of S and M. In order to evaluate the matrix elements of $\hat{P}_{AB}^{S}$, we recouple the angular momenta to yield an eigenbasis of $\hat{\mathbf{S}}_{AB} = \hat{\mathbf{S}}_A + \hat{\mathbf{S}}_B$, $\hat{\mathbf{S}}_C$, and $\hat{\mathbf{S}}$:

$$\left|((S_A,S_C)S_{AC},S_B)S,M\right\rangle = \sum_{S_{AB}}\left|(S_C,(S_A,S_B)S_{AB})S,M\right\rangle\left\langle(S_C,(S_A,S_B)S_{AB})S\right|\left((S_A,S_C)S_{AC},S_B)S\right\rangle, \tag{S.8}$$

with the recoupling coefficient given in terms of Wigner 6-j symbol by:

$$\left\langle(S_C,(S_A,S_B)S_{AB})S\right|\left((S_A,S_C)S_{AC},S_B)S\right\rangle = (-1)^{S_A+S_B+S_C+S}\sqrt{(2S_{AB}+1)(2S_{AC}+1)}\begin{Bmatrix}S_C & S_A & S_{AC}\\ S_B & S & S_{AB}\end{Bmatrix}. \tag{S.9}$$

Combining eq. (S.6) and (S.8), we may thus write

$$\hat{\rho}(t) = \frac{1}{\mathrm{Tr}\left[\hat{P}_{\mathrm{AB}}^{\mathrm{S}}\right]} \sum_{S_{AC}=J-\frac{1}{2}}^{J+\frac{1}{2}} \sum_{S=\left|S_{AC}-\frac{1}{2}\right|}^{S_{AC}+\frac{1}{2}} \sum_{S_{AB}=0}^{1} \sum_{M=-S}^{S} A^2(S_{AC}) B(S_{AB}) \left| \left\langle (S_C, S_{AB}) S \right| (S_{AC}, S_B) S \right\rangle \right|^2$$
$$= \frac{1}{\mathrm{Tr}\left[\hat{P}_{\mathrm{AB}}^{\mathrm{S}}\right]} \sum_{S_{AC}=J-\frac{1}{2}}^{J+\frac{1}{2}} \sum_{S=\left|S_{AC}-\frac{1}{2}\right|}^{S_{AC}+\frac{1}{2}} \sum_{S_{AB}=0}^{1} (2S+1) A^2(S_{AC}) B(S_{AB}) \left| \left\langle (S_C, S_{AB}) S \right| (S_{AC}, S_B) S \right\rangle \right|^2, \quad \text{(S.10)}$$

where

$$B(S_{AB}) = \left\langle (S_C, S_{AB}) S, M \right| \hat{P}_{\mathrm{AB}}^{\mathrm{S}} \left| (S_C, S_{AB}) S, M \right\rangle$$
$$= \frac{1}{2}\left(\tfrac{1}{2} + S_A(S_A+1) + S_B(S_B+1) - S_{AB}(S_{AB}+1) \right), \quad \text{(S.11)}$$

using the M-independence of the summands. It is clear that for the singlet initial configuration $B(S_{AB})$ vanishes except for the singlet basis, i.e. $S_{\mathrm{AB}} = 0$, for which $B(0) = 1$. This condition also implies that non-zero contributions can only result from $S = S_{\mathrm{C}} = J$. As a consequence,

$$\hat{\rho}(t) = \frac{(2J+1)}{\mathrm{Tr}\left[\hat{P}_{\mathrm{AB}}^{\mathrm{S}}\right]} \sum_{S_{AC}=J-\frac{1}{2}}^{J+\frac{1}{2}} A^2(S_{AC}) \left| \left\langle (S_C = J, S_{AB} = 0) S = J \right| (S_{AC}, S_B = \tfrac{1}{2}) S = J \right\rangle \right|^2$$
$$= \sum_{S_{AC}=J-\frac{1}{2}}^{J+\frac{1}{2}} A^2(S_{AC}) (2S_{AC}+1) \left| \begin{Bmatrix} J & \frac{1}{2} & S_{AC} \\ \frac{1}{2} & J & 0 \end{Bmatrix} \right|^2. \quad \text{(S.12)}$$

Here, we have used $\mathrm{Tr}\left[\hat{P}_{\mathrm{AB}}^{\mathrm{S}}\right] = 2J+1$. The required 6-j symbol is

$$\left| \begin{Bmatrix} J & \frac{1}{2} & S_{AC} \\ \frac{1}{2} & J & 0 \end{Bmatrix} \right|^2 = \frac{1}{2(2J+1)}, \quad \text{(S.13)}$$

which allows us to evaluate the sum. Simple algebraic manipulation eventually yields eq. (6) for $k_{\mathrm{C}}^{+} = 0$.

An analogous approach can be used to derive the singlet probability in the subspace of spin A and B (eq. (8)). In particular,

$$\mathrm{Tr}\left[\hat{P}_{\mathrm{AB}}^{\mathrm{S}} \hat{\rho}(t)\right] = \frac{1}{\mathrm{Tr}\left[\hat{P}_{\mathrm{AB}}^{\mathrm{S}}\right]} \mathrm{Tr}\left[\hat{P}_{\mathrm{AB}}^{\mathrm{S}} \exp\left(-\tfrac{1}{2} k_{\mathrm{C}}^{-} \hat{P}_{\mathrm{AC}}^{-}\right) \hat{P}_{\mathrm{AB}}^{\mathrm{S}} \exp\left(-\tfrac{1}{2} k_{\mathrm{C}}^{-} \hat{P}_{\mathrm{AC}}^{-}\right)\right]$$
$$= \sum_{S_{AC}=J-\frac{1}{2}}^{J+\frac{1}{2}} \sum_{S'_{AC}=J-\frac{1}{2}}^{J+\frac{1}{2}} A(S_{AC}) A(S'_{AC}) \left| \left\langle (S_C = J, S_{AB} = 0) S = J \right| (S_{AC}, S_B = \tfrac{1}{2}) S = J \right\rangle \right|^2 \cdot$$
$$\left| \left\langle (S_C = J, S_{AB} = 0) S = J \right| (S'_{AC}, S_B = \tfrac{1}{2}) S = J \right\rangle \right|^2, \quad \text{(S.14)}$$

from which eq. (8) follows.
